# Third Palatal Rugae as Stable Landmarks for Intraoral Models' Superimposition in Extraction Cases: A Retrospective Cohort Study

**DOI:** 10.1111/ocr.70078

**Published:** 2025-12-25

**Authors:** Carolina Morsani Mordente, Lorenna de Souza Dores, Dauro Douglas Oliveira, Juan Martin Palomo, Bernardo Quiroga Souki, Rodrigo Villamarim Soares

**Affiliations:** ^1^ Pontifical Catholic University of Minas Gerais Belo Horizonte Brazil; ^2^ Department of Orthodontics, School of Dentistry Case Western Reserve University Cleveland Ohio USA

**Keywords:** cone‐beam computed tomography, diagnostic imaging, orthodontic tooth movements, palate rugae, tooth extraction

## Abstract

**Objectives:**

To validate the third palatal rugae as stable reference landmarks for the superimposition of maxillary digital models in premolar extraction cases.

**Materials and Methods:**

Maxillary intraoral scanning digital models of 22 extraction patients, obtained before (T0) and after approximately 4 months of incisor retraction (T1), were superimposed using the automated best‐fit of the third palatal rugae and palatine raphe. Voxel‐based registration of CBCT scans served as the gold standard. Point‐to‐point displacements between T0 and T1 of the central incisors and cuspids were calculated for both methods.

**Results:**

The mean differences (mismatch) between methods in the combined vertical and horizontal perspectives of the central incisors were 0.08 mm (right) and 0.12 mm (left), respectively. For the cuspids, the mean 2D differences were 0.02 mm (right) and 0.07 mm (left). The pure horizontal displacement differences of the maxillary central incisors were 0.13 mm (right) and 0.04 mm (left). For the cuspids, the differences were: 0.05 mm (right) and 0.03 mm (left). No statistically significant differences were found between the intraoral‐scan and CBCT measurements for either the central incisors or cuspids after maxillary superimpositions. The differences between the two methods were smaller than 0.2 mm in all comparisons.

**Conclusions:**

Digital superimposition of the third palatal rugae of models obtained with intraoral scans is a valid method for the 3D evaluation of the short‐term (4‐month) dental changes in the anterior region of the maxilla in cases of premolar extraction followed by incisor retraction.

**Trial Registration:**

ClinicalTrials.gov: NCT03089996 and NCT05281588

## 1 Introduction

Maxillary superimposition of orthodontic records from different timepoints enables clinicians to assess treatment outcomes, monitor changes in dentofacial structures and make informed decisions regarding patient care [1]. Accurate superimposition methods are essential for tracking changes over time, facilitating the evaluation of treatment effectiveness and stability. Traditionally, cephalograms have been the gold standard for maxillary superimposition in dentistry [2]. However, over the past two decades, cone beam computed tomography (CBCT) has emerged as a highly accurate tool, providing accurate three‐dimensional (3D) representations of bone structures. The development of voxel‐based registration has made CBCT a valid method for superimposition of maxillofacial structures [3, 4]; and user‐friendly software now supports its reliable application [5]. Despite its advantages, concerns regarding the clinical implications of CBCT radiation exposure, particularly in cases involving multiple assessments during treatment, should be considered [6].

An alternative to CBCT for maxillofacial superimposition is intraoral scanning, which has gained popularity in orthodontic clinical practice [7]. Intraoral scanners generate virtual dental models that can be volumetrically registered, offering a noninvasive, cost‐effective and patient‐friendly method for maxillary superimposition [8, 9]. Additionally, intraoral scanning provides a radiation‐free alternative that has shown promise in various dental applications [10, 11]. Reports have demonstrated high accuracy and reliability of palatal rugae superimposition using digitised cast models, even in cases involving slow [12] or rapid maxillary expansion [13], as well as teeth extractions with significant incisors' retraction [14, 15], which may result in anatomical changes in the palatal soft tissue and rugae.

However, other studies have highlighted some limitations in the use of palatal rugae for superimposition. Pazera and Gkantidis [16] described that, although the anteroposterior component of palatal rugae superimposition is reliable, the vertical component does not exhibit the same level of reliability compared to cephalometric parameters. Ziar et al. [17] also reported that palatal rugae stability is compromised following extractions from a 3D perspective. More recently, Makrygiannakis et al. [18] reported that the palatal rugae shape changes during orthodontic treatment, with extraction cases exhibiting a more pronounced effect than non‐extraction cases. Furthermore, a systematic review with meta‐analysis by Gupta et al. [19] concluded that, despite the stability of palatal rugae during orthodontic incisor retraction, future studies should address potential examiner bias in visual inspection of rugae and evaluate the use of automated computational tools to reduce such methodological limitations.

Given these considerations, the present study was undertaken to assess the validity of automated palatal rugae superimposition of intraoral digital models for 3D evaluation of orthodontic changes in cases involving maxillary premolar extraction followed by incisors' retraction. It was hypothesised that the third palatal rugae automated superimposition method, using intraoral scanning models, provides a valid alternative for maxillary superimposition even in scenarios that involve substantial anatomical changes in the anterior maxilla, such as significant incisor retraction. In principle, this method could ensure accurate monitoring of dentofacial changes during orthodontic treatment.

## 2 Material and Methods

### 2.1 Study Design and Sample

This study followed the STROBE statement for transparent reporting of retrospective cohort studies [20]. The investigation was approved by the institutional review board of the Pontifical Catholic University of Minas Gerais (Belo Horizonte, Brazil) under protocol #87658218.2.0000.5137.

The present investigation is a secondary analysis of data obtained from a previously published randomised clinical trial (RCT) conducted at the same institution between February 2016 and December 2019. The RCT included patients requiring orthodontic retraction of the maxillary incisors with multibracket edgewise mechanics after bilateral first premolar extractions. Its primary outcomes and full methodological details, including screening, inclusion and randomisation procedures have been reported elsewhere [21]. All participants provided written informed consent prior to enrollment in the randomised clinical trial.

The primary objective of the current analysis was to assess the validity of intraoral scanning volumetric data registered at the third palatal rugae, compared to CBCT voxel‐based registration in the anterior cranial fossa, in cases of bilateral first premolar extraction and orthodontic incisor retraction. Similarity between the tooth displacement measurements obtained with the two methods would support the use of the third palatal rugae for superimposing maxillary intraoral models.

An a priori sample size calculation was performed using G*Power 3.1 (Heinrich Heine University, Düsseldorf, Germany). Based on preliminary data, a pooled standard deviation of 0.12 mm and a minimum detectable difference of 0.13 mm were assumed for the primary outcome (difference in tooth displacement between methods), resulting in an effect size (Cohen's *d*) of 1.08. With a two‐tailed paired *t*‐test, *α* = 0.05 and power (1−*β*) = 0.95, the required sample size was 21 individuals. The CBCT scans had a spatial resolution of 0.3 mm (voxel size), defining the approximate lower threshold for detecting differences in three‐dimensional measurements.

The final sample comprised 22 patients (13 male, 9 female; age range: 16–40 years; mean age: 24.64 ± 7.21 years), all drawn from the RCT database. Record selection for the current analysis followed the eligibility criteria described in the RCT publication and applied the following inclusion criteria: (1) age ≥ 16 years with skeletal maturity confirmed by the cervical vertebral maturation method (CVM 6) [22], (2) indication for orthodontic maxillary incisor retraction, (3) presence of all permanent maxillary teeth except third molars and (4) availability of CBCT scans at T0 (immediately before incisor retraction) and at T1 (4 months after incisor retraction). Exclusion criteria were: (1) systemic diseases or medications affecting bone biology, (2) pregnancy, (3) poor oral hygiene, (4) previous orthodontic treatment, (5) bone loss, (6) active periodontitis, (7) smoking, (8) craniofacial syndromes or cleft palate, (9) severe crowding, (10) severe Class II malocclusion (ANB > 7°, overjet > 10 mm) and (11) hyperdivergency (SNGoGn > 38°).

### 2.2 Image Acquisition

Intraoral scanning was performed using the Trios 3 (3Shape, Copenhagen, Denmark). The full palate extension was included in the maxillary arch for all patients, and files were exported in ‘.stl’ format.

CBCT scans were acquired using the i‐CAT (Imaging Sciences International, Hatfield, Pennsylvania, USA) with a field of view (FOV) of 16 × 22 cm, voxel size of 0.3 × 0.3 × 0.3 mm, 36.90 mA, 120 kV and 40 s of exposure. Patients were instructed to maintain maximum bite pressure during the CBCT acquisition. Scans were obtained on the same day as the intraoral scans at both T0 and T1.

### 2.3 Image Analysis

**FIGURE 1:**
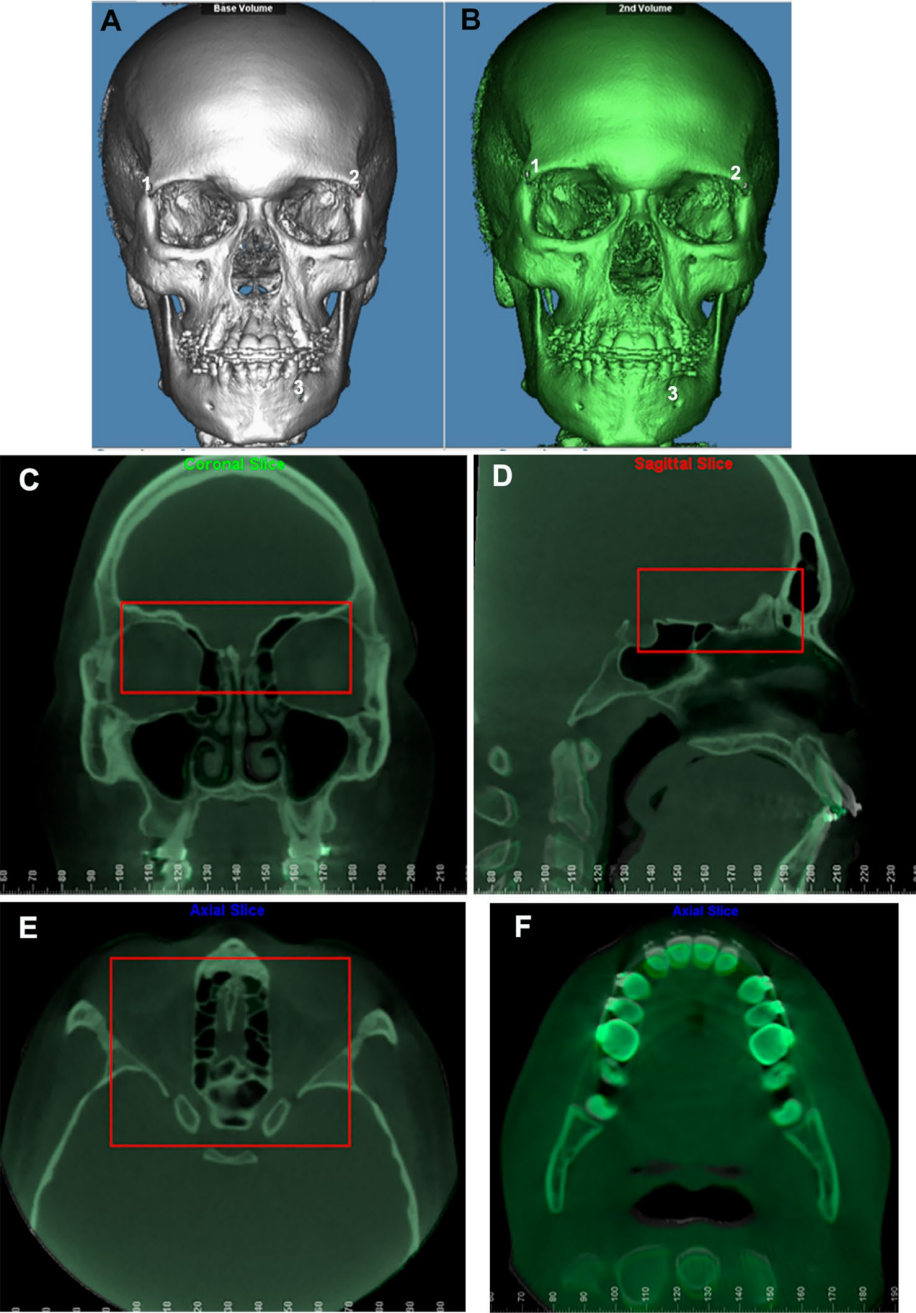
Voxel‐based superimposition of CBCT scans on the cranial base. (A) Approximation of the initial (white) and (B) final (green) CBCTs in the Dolphin Imaging software by the selection of three points: (1) right frontozygomatic suture; (2) left frontozygomatic suture; (3) left mental foramen; (C, D, E) Selection of the reference area at the cranial base for the automatic superimposition of the initial and final CBCTs by the voxel method in Dolphin Imaging software (red box) in front view (C), lateral view (D), top view (E); (F) visualisation of the initial and final CBCTs superimposed at the crown level of the maxillary incisors.

Head orientation of the T0 CBCT scans was standardised within the same Cartesian coordinate system [23] using VistaDent 3D software (Dentsply GAC, York, PA, USA). The T1 scans were manually aligned to the T0 oriented scans and then volumetrically superimposed using a cranial base voxel‐based method (Dolphin Imaging software, Chatsworth, CA, USA), which is a fast, accurate and reliable technique [5]. The ‘auto superimposition’ tool was used to select a target area at the cranial base for automatic superimposition (Figure 1). The accuracy and quality of the superimposition were verified through multiplanar sections (sagittal, coronal and axial) by one trained operator (C.M.M.).

**FIGURE 2:**
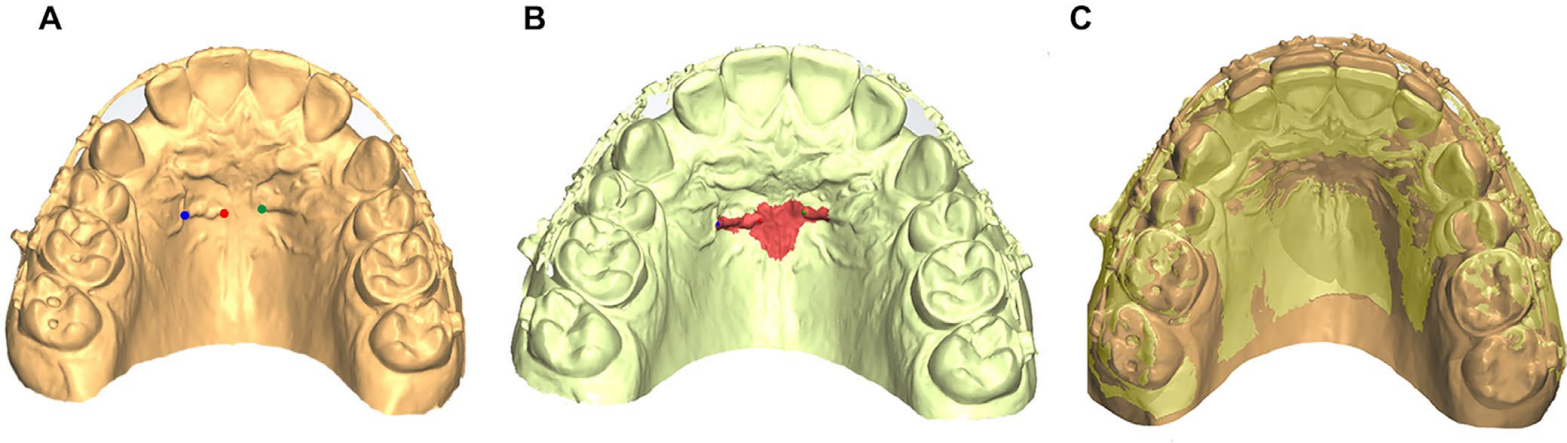
Three‐point and 1‐surface registration method at the third palatal rugae using Ortho Analyser software. (A) Selection of three landmarks: The most medial aspect of the right (red) and left (green) third rugae, and a reproducible site at the center of the right third ruga (blue). (B) Definition of the palatal surface using the 1‐mm radius brush tool, extending laterally along the third rugae, 1.5 mm anteriorly and 6 mm posteriorly along the palatal raphe. (C) Superimposition of T0 and T1 models.

Subsequently, T0 intraoral digital models were manually merged with the oriented T0 CBCT scans using the best‐fit method in VistaDent 3D software. The oriented T0 models were then exported in ‘.stl’ format for automated superimposition in Ortho Analyser (3Shape A/S, Copenhagen, Denmark) using the 3‐point and 1‐surface registration method based on the palatal rugae. Three reproducible landmarks were placed on the palatal rugae: the most medial aspect of the right and left third rugae and the center of the right third rugae. In the same sequence, the palatal surface surrounding the rugae was painted with the brush tool (1‐mm radius; 10 consecutive mouse clicks corresponding to 10 connected brush strokes), extended laterally along the third rugae, anteriorly 1.5 mm and posteriorly 6 mm from the third rugae along the palatal raphe. A secondary screen displaying the T0 model with pre‐marked points and surface was used to guide the selection of corresponding T1 landmarks and surface, ensuring reproducibility. Once both the landmarks and surface region were defined, the software internally executed the computational process in two stages: (1) a rigid superimposition (translation and rotation, without scaling) based on the three points, followed by (2) refinement with the iterative closest point algorithm applied to the triangular meshes of the selected region [21, 24]. Figure 2 illustrates the identification of the three reference points and the palatal surface boundaries used for registration. For illustrative purposes, the landmarks were enlarged and colour‐coded in the figure to facilitate visualisation; however, during the computational procedure, a very small point was selected in Ortho Analyser to ensure precise registration.

### 2.4 Quantitative Evaluation of Teeth Displacements

The agreement between the displacements of the incisors and cuspids from T0 to T1, as measured using both CBCT and intraoral scanning methods, would indicate the validity of the palatal rugae superimposition technique for precise matching of maxillary records during incisor retraction. Disagreement in the measured displacements would suggest that the palatal rugae underwent anatomical changes during orthodontic treatment and could not be reliably used for digital model superimposition.

The point‐to‐point displacement of the midpoint of the incisal border of the central incisors and the cuspids between T0 and T1 was measured to assess 3D positional changes in the maxillary arch using Ortho Analyser software. Displacements measured included: (a) horizontal displacements (anterior–posterior for the incisors and right–left for the cuspids), relative to the axial plane and (b) the combination of the vertical displacement with either the anterior–posterior (incisors) and right–left (cuspids), named as 2D distance.

**FIGURE 3:**
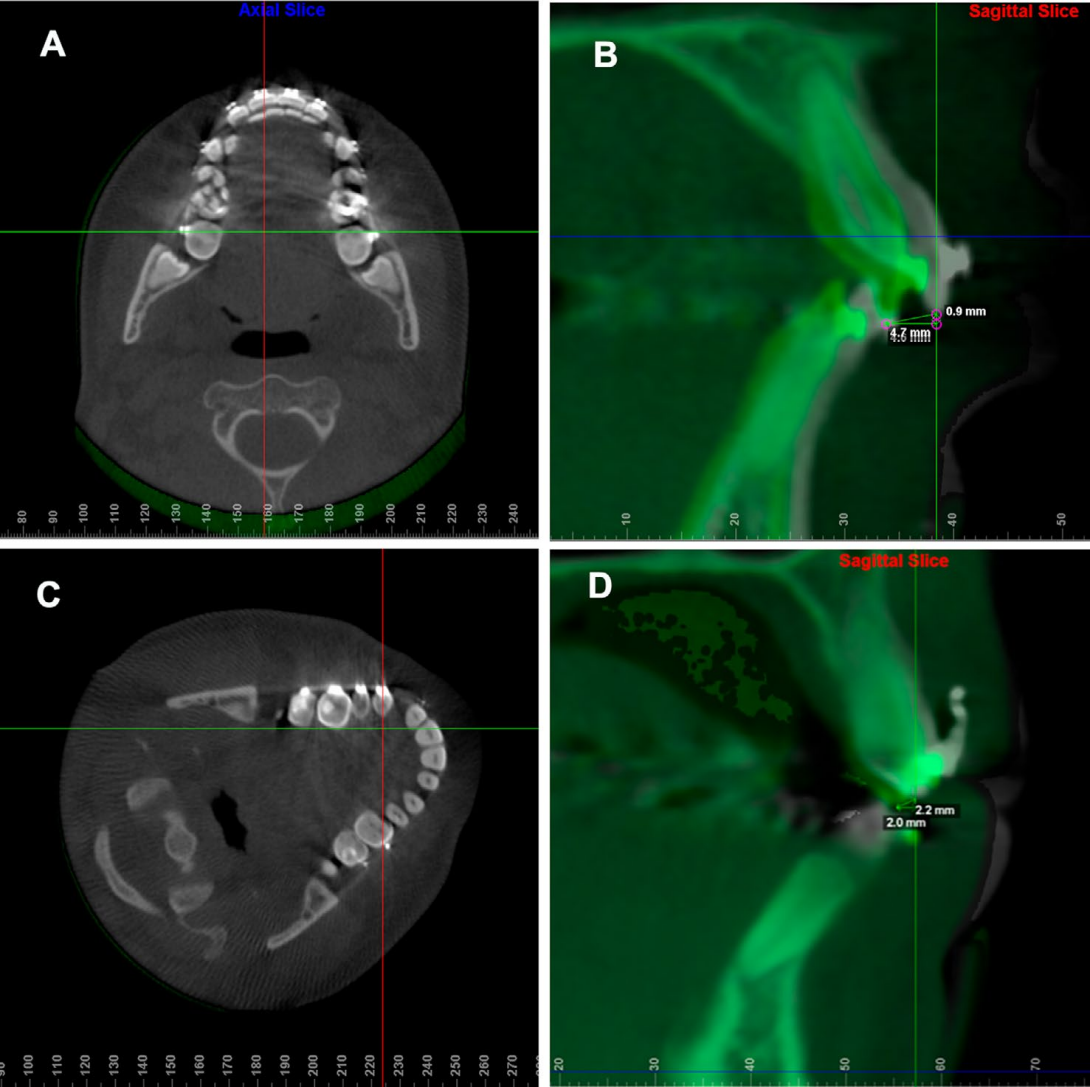
CBCT measurements using Dolphin Imaging software. (A) in the axial view, selection of the cut that passes through the center of the incisal edge of the maxillary right central incisor (red line) at T0; (B) in the sagittal view, measurement of 2D and horizontal displacement from the lowest point of the incisal border from T0 (grey) to T1 (green); (C) in axial view, after rotating the initial CBCT, selection of the slice that passes through the center of the crown of the maxillary right cuspid (red line); (D) in the sagittal view, measurement of 2D (combination of horizontal and vertical displacement) and the pure horizontal displacement of the lowest point of the incisal border from T0 (grey) to T1 (green).

The Dolphin Imaging software was used for CBCT measurements. Using the axial slice passing through the center of the central incisors at T0, a line was drawn through the midpoint of the incisal border, establishing the mid‐sagittal plane (Figure 3A). The shortest distance between the lowest points of the incisal border at T0 and T1 in the sagittal section was measured to calculate the 2D displacement, which included both vertical and anterior–posterior components. A parallel line through the lowest point of the incisal border was used to calculate the pure horizontal displacement (Figure 3B). The same measurement technique was applied to both the right and left central incisors.

For cuspids, similar measurements were conducted, with the axial section rotated to position the mid‐sagittal plane at a right angle to the midpoint of the cuspids, and passing through the center of the crown (Figure 3C,D). The same method was applied to both right and left cuspids. Thus, a 3D perspective was covered by the current methodology. Once again, sagittal, vertical and transverse mismatch of the model's superimposition would indicate failure in the validity of the third palatal rugae as an anatomical reference.

**FIGURE 4:**
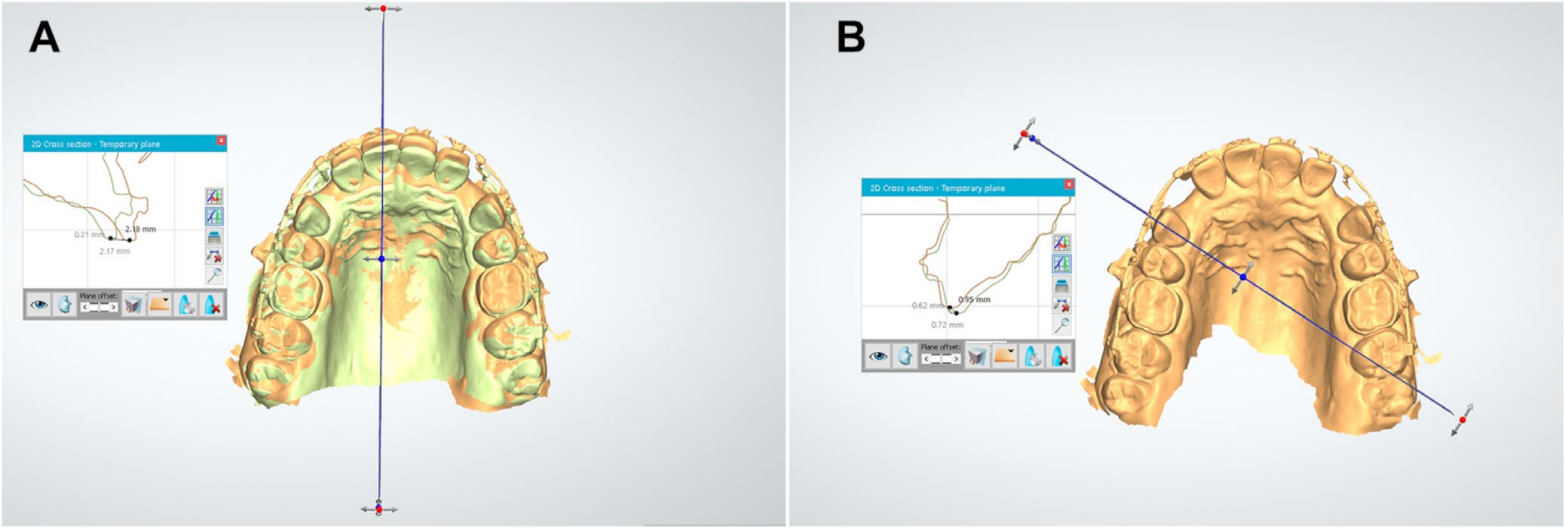
Digital models measurements using the Ortho Analyser software. (A) creation of a plane perpendicular to the ground that intersects the center of the incisal edge of the central incisor at T0 (right) and measurement of the 2D and horizontal displacements of the lower point of the incisal edge of the central incisor (left); (B) creation of a plane perpendicular to the ground that intersects the center of the cuspid at T0 (right) and measurement of the 2D and horizontal displacements of the lower point of the incisal edge of the cuspid (left).

Digital maxillary models were assessed using the ‘cross‐section’ feature of Ortho Analyser software. A plane was set parallel to the ground in the occlusal view of the T0 models, crossing the central incisor's incisal edge in the middle. The projection of this plane in the 2D section revealed the outline of the central incisors at T0 and T1, facilitating the identification of the lowest point on the incisal borders, enabling the automated calculation of their 2D and pure horizontal displacements (Figure 4A). The same method was used for both right and left central incisors and cuspids. For cuspid displacement measurements, the ‘cross‐section’ tool established a perpendicular plane to the ground through the center of the crown of the teeth at T0 (Figure 4B).

### 2.5 Statistical Analysis

All measurements were performed by a single examiner, calibrated and trained by the senior researcher to ensure consistency, and conducted blindly with respect to patient identification. Intra‐examiner reliability was assessed by repeating the measurements of seven randomly selected pairs of intraoral models and CBCTs after a two‐week interval under identical conditions. Reliability was evaluated using intraclass correlation coefficients (ICCs) obtained with a two‐way random‐effects model for average measures [ICC(2,k)] and the Dahlberg formula.

Age and sex were evaluated as potential confounding variables in exploratory analyses, but since no statistically significant associations with displacement values were found, no further adjustments were applied in the final statistical comparisons.

The D'Agostino‐Pearson test confirmed that the data followed a normal distribution (*p* > 0.05 for all variables) and means and standard deviations were calculated for all variables. Comparisons between methods were performed using paired *t*‐tests to assess displacement measurements between T0 and T1 records from digital model and CBCT pairs. Correlation analysis was performed using Pearson's correlation coefficient (*r*) to assess the relationship between the incisor retraction rate and the agreement between the two methods.

A significance level of 5% was set. Statistical analysis was performed using GraphPad Prism 5.0 (GraphPad Software, San Diego, California, USA).

## 3 Results

Table 1 presents the reliability analysis. The ICC demonstrated excellent intra‐examiner reliability within each method, ranging from 0.97 to 0.99 for digital model pairs and from 0.92 to 0.99 for CBCT pairs. Method error, as assessed by the Dahlberg formula, varied between 0.061 and 0.436 mm, indicating that the measurement error was minimal and well within clinically negligible limits.

**TABLE 1:** Method error quantification: Dahlberg formula and ICC values.

	Intraoral model		CBCT	
	Dahlberg	ICC	Dahlberg	ICC
Pure horizontal displacement				
Right central incisor	0.089	0.996	0.272	0.962
Left central incisor	0.168	0.991	0.411	0.924
Right cuspid	0.061	0.996	0.371	0.798
Left cuspid	0.086	0.991	0.246	0.911
2D displacement				
Right central incisor	0.101	0.996	0.281	0.961
Left central incisor	0.155	0.992	0.436	0.919
Right cuspid	0.081	0.994	0.383	0.794
Left cuspid	0.181	0.967	0.289	0.881

Abbreviations: 2D, two‐dimensional; CBCT, cone beam computed tomography; ICC, intra class correlation coeficient.

A total of 22 patients (44 central incisors and 44 cuspids) were analysed. No exclusions were performed during the analytical stage of the investigation.

Table 2 presents the mean, standard deviation and 95% confidence intervals values for the pure horizontal displacement and 2D displacement of the maxillary central incisors and cuspids. The mean 2D differences between methods (i.e., discrepancies in maxillary central incisor measurements between intraoral scans and CBCT) were 0.08 mm on the right side (2.84 mm vs. 2.76 mm, *p* = 0.716) and 0.12 mm on the left side (2.80 mm vs. 2.68 mm, *p* = 0.710). For the cuspids, the mean 2D differences were: 0.02 mm (1.37 mm vs. 1.35 mm, right, *p* = 0.917) and 0.07 mm (1.37 mm vs. 1.44 mm, left, *p* = 0.674).

**TABLE 2:** Pure horizontal displacements (AP for maxillary central incisors; MD for cuspids) and combined 2D displacements (vertical plus AP for incisors; vertical plus MD for cuspids).

	Intraoral model						CBCT						*p*
	Min (mm)	Max (mm)	Incisor's retraction		Mean ± SD	95% CI	Min (mm)	Max (mm)	Incisor's retraction		Mean ± SD	95% CI	
			< 2 mm (*n*)	> 4 mm (*n*)					< 2 mm (*n*)	> 4 mm (*n*)			
Pure horizontal displacement													
Right central incisor	1.76	4.65	2	1	2.61 ± 0.99	2.11–3.05	1.90	4.60	1	1	2.74 ± 0.90	2.34–3.14	0.653
Left central incisor	1.41	4.80	2	1	2.66 ± 1.07	2.19–3.13	1.90	4.70	1	1	2.70 ± 0.88	2.31–3.09	0.894
Right cuspid	0.07	2.39	NA	NA	1.21 ± 0.68	0.91–1.51	0.70	2.30	NA	NA	1.26 ± 0.55	1.02–1.50	0.791
Left cuspid	0.33	2.66	NA	NA	1.27 ± 0.61	1.00–1.54	0.10	1.80	NA	NA	1.24 ± 0.58	0.98–1.50	0.869
2D displacement													
Right central incisor	1.77	4.91	2	1	2.76 ± 0.98	2.33–3.19	2.00	4.60	0	1	2.84 ± 0.86	2.46–3.22	0.716
Left central incisor	1.41	5.07	2	1	2.68 ± 0.96	2.25–3.11	2.00	4.70	0	1	2.80 ± 0.86	2.42–3.18	0.710
Right cuspid	0.30	2.50	NA	NA	1.35 ± 0.69	1.04–1.66	0.60	2.40	NA	NA	1.37 ± 0.56	1.12–1.62	0.917
Left cuspid	0.40	2.70	NA	NA	1.44 ± 0.60	1.17–1.71	0.20	2.50	NA	NA	1.37 ± 0.48	1.16–1.58	0.674

*Note:* Values in milimiters. *p* value paired *t*‐test.

Abbreviations: 2D, two‐dimensional; AP, anteroposterior; CBCT, cone beam computed tomography; CI, confidence interval; Max, maximum; MD, mesiodistal; Min, minimum; NA, not applicable; SD, standard deviation.

The horizontal displacements of the maxillary central incisors and the differences between methods were: 0.13 mm (2.74 mm vs. 2.61 mm, right, *p* = 0.653) and 0.04 mm (2.70 mm vs. 2.66 mm, left, *p* = 0.894). For the cuspids, the differences were: 0.05 mm (1.26 mm vs. 1.21 mm, right, *p* = 0.791) and 0.03 mm (1.24 mm vs. 1.27 mm, left, *p* = 0.869). The paired *t*‐test revealed no statistically significant differences between the intraoral scan and CBCT measurements for either the incisors or cuspids after maxillary superimpositions. Therefore, differences between the two methods were smaller than 0.2 mm in all comparisons, a value below the spatial resolution of the CBCT scans used in this study.

The amount of incisor retraction ranged from 1.41 to 5.07 mm across both measurement methods (intraoral scans and CBCT) and across both 2D and horizontal directions. A stratified analysis showed that retractions < 2 mm occurred in two individuals, while retractions > 4 mm were observed in at least one case for nearly all variables. Notably, a correlation analysis between the average retraction magnitude and the absolute difference between CBCT and intraoral scan measurements revealed a moderate negative correlation (*r* = −0.39), suggesting that greater retractions were not associated with increased variability between the two methods.

Minor canine movements between T0 and T1 were due to the binding effect of the heavy stainless‐steel wire following the previous stage of canine retraction, as well as to the en‐masse maxillary arch retraction anchored in skeletal temporary devices.

## 4 Discussion

This study analysed 22 orthodontic patients (13 male and nine female; mean age 24.6 ± 7.2 years) who had achieved skeletal maturity, confirmed by CVM stage 6. All patients underwent bilateral maxillary first premolar extractions followed by incisor retraction and were evaluated over a short observational interval, with a mean duration of 4.1 months between pre‐ and post‐treatment records (T0–T1). Within this context, the present investigation compared linear tooth displacement measurements obtained through intraoral scan–based superimposition and CBCT‐based voxel registration. The findings demonstrated that intraoral scanning could achieve reliable automated three‐dimensional maxillary superimposition using the third palatal rugae as an anatomical reference, even in cases involving substantial orthodontic movement of the incisors.

Digital models provided linear data, allowing an accurate and comprehensive assessment of dental changes in all spatial dimensions because it was observed that the measurements of orthodontic displacement of the maxillary central incisors and cuspids did not present statistically significant differences between the two methods of maxillary superimposition: intraoral digital models and CBCT scans. The very small differences observed between the two methods remained within the expected measurement error (< 0.3 mm) and were of no clinical significance, providing evidence that STL‐based superimposition at the level of the palatal rugae may be a clinically acceptable alternative in clinically similar contexts. Therefore, the 3D automated superimposition technique enabled a precise analysis of the effects of orthodontic treatment, which is critical for occlusal analysis.

The superimposition of digitalized cast models for dental evaluations has been the subject of many previous investigations regarding the validation of the superimposition of digital models [10, 25], but recently, with the increased use of intraoral scanning, a new era in orthodontics was initiated [9, 26]. Cha et al. [2] used an entire palate area to superimpose digital models on patients getting maxillary premolar extractions and orthodontic treatment. Their results demonstrated the efficacy of a specific palate region for precise assessment of dental movements in both horizontal and vertical dimensions. However, it is essential to highlight that their method primarily relied on cephalograms for superimposition, and this approach has inherent limitations. Notably, cephalograms do not consider the three planes of space, which is a fundamental limitation when attempting to accurately assess complex dental changes in three‐dimensional contexts. The present study used a novel investigative approach by comparing the reliability of digital models generated directly by intraoral scanning with the superimposition of CBCT scans, the gold standard of the true skeletal base. It is important to note that to the best of our knowledge, the current study was the first to use valid voxel‐based cranial base registration as a reference point for the 3D superimposition of dental models. Despite the cranial base use for the maxilla analysis, it is important to mention that the individuals in the current sample did not present residual facial growth, and the interval between T0 and T1 was only a few months. Therefore, the anterior cranial fossa can be used as a true positional reference for the maxilla and its teeth.

Gupta et al. [19] performed a systematic review on the reliability of palatal rugae as a tool forpersonal identification following orthodontic treatment and concluded that digital automation of identification of the rugae is necessary to eliminate the subjective bias of the human eye. Thus, the inclusion of automated computational systems that match the two time‐point scans, as performed in the current study, enhances the reliability of the maxillary surface registration. Comfort and safety can be offered to patients with the use of intraoral scanning [9], instead of traditional impression methods to obtain cast models. Convenience and time savings for clinicians can result from the use of automated 3D superimposition of digital models. Therefore, this approach may represent a clinically useful addition to the orthodontic armamentarium, as the present findings suggest it can provide a reasonable estimation of treatment outcomes with reduced invasiveness and increased practicality.

These results confirm that the third palatal rugae remained stable, even after premolar extractions and significant incisor retraction, diverging from earlier reports suggesting potential distortions in palatal morphology following such treatments. In contrast to studies by Zhao et al. [27], Ziar et al. [17] and Makrygiannakis et al. [18] which observed morphological changes over longer intervals (12–30 months), our study involved a shorter treatment duration and used intraoral digital scans rather than alginate impressions. The latter can introduce distortions in soft tissue details, potentially affecting rugae assessment. Furthermore, by including only skeletally mature patients (CVM stage 6) and limiting the interval between T0 and T1 to a few months, growth‐related changes were minimised. These methodological differences likely account for the divergence in findings regarding rugae stability across studies. Intraoral scans are routinely acquired during orthodontic treatment, and most are already integrated into digital workflows using planning software such as ClinCheck (Align Technology), OrthoAnalyzer (3Shape), Maestro 3D and Blue Sky Plan. The ability to perform superimpositions directly from STL files streamlines the process and eliminates the need for additional imaging, such as CBCT scans, when hard tissue structures are not the focus of the analysis. This approach can reduce radiation exposure, lower overall costs and accelerate assessment timelines, especially in cases where tooth movement monitoring or treatment refinement is needed. Furthermore, using intraoral scans enhances patient comfort and aligns with the growing emphasis on digital orthodontics and personalised care.

There are some limitations in the present study. The retrospective design introduces the potential for selection bias, as it relies on previously collected data. Additionally, the sample included patients with varying characteristics, such as different ethnicities, facial types, palatal thicknesses, rugae depths and malocclusions, which may introduce confounding bias. The present sample did not evaluate such variabilities. Prior research [7] has shown that rugae characteristics can differ among populations, which may influence the generalisability of these findings to broader or more diverse clinical settings. Although this study did not control for demographic or morphological confounders, future investigations may benefit from stratified analyses to assess their potential influence on superimposition accuracy.

Another limitation is the use of a single software for maxillary superimposition, as testing alternative applications could yield different results. The results may reflect software‐specific processing, as the superimpositions were conducted using Ortho Analyser. Variations in algorithm design, mesh resolution and surface registration parameters between different software platforms could introduce bias and limit reproducibility across clinical systems. To address these issues, a prospective study with a more specific patient group is recommended, allowing for the evaluation of the impact of various anthropometric variables on the results. Furthermore, the use of open‐source software capable of automating the superimposition process could enhance the study's accuracy and reproducibility.

Moreover, the relatively small sample size, which may constrain generalisability, can be raised as a limitation. However, a post hoc power analysis based on the observed effect size (Cohen's *d* = 1.08) confirmed that the sample of 22 patients provided sufficient statistical power (99.8%) to detect meaningful differences between the two superimposition methods. Additionally, although multiple comparisons were performed across tooth types and displacement directions, all tests were conceptually linked to a single hypothesis regarding the validity of rugae‐based registration. Therefore, we opted not to apply a formal correction for multiple comparisons to avoid an undue increase in Type II error. Nevertheless, we acknowledge that the absence of such correction may slightly elevate the risk of false‐positive findings, and results should be interpreted within this context.

In summary, this study addressed a critical gap in the literature by providing three‐dimensional, CBCT‐referenced evidence on the accuracy of intraoral digital model superimposition using the third palatal rugae as a landmark. By anchoring comparisons in a validated cranial base reference, the study enabled a robust assessment of superimposition accuracy. The results indicate that this approach can closely replicate outcomes obtained through high‐resolution volumetric imaging and may serve as a predictable alternative to previously established palate‐based methods, with the added benefit of overcoming the limitations of cephalograms. Integrating intraoral scanning technology into treatment analysis allows orthodontists to assess three‐dimensional dental changes throughout treatment with greater efficiency, reduced invasiveness and improved patient comfort. Further research should explore broader clinical scenarios to confirm these findings and expand the use of digital treatment planning and assessment in orthodontics.

## 5 Conclusions

The superimposition of the third palatal rugae of models obtained with intraoral scans is a valid method for the 3D evaluation of dental changes in the anterior region of the maxilla in cases of premolar extraction followed by the retraction of the incisors.

## Author Contributions

All authors contributed to the conception or design of the work. C.M.M. collected the data. C.M.M., L.S.D., D.D.O., J.M.P., B.Q.S. and R.V.S. performed data analysis and interpretation, C.M.M. drafted the article, B.Q.S. and R.V.S. critically reviewed the article and all authors approved the final version to be published.

## Ethics Statement

This study was approved by the institutional review board of the Pontifical Catholic University of Minas Gerais (Belo Horizonte, Brazil) under protocol #87658218.2.0000.5137.

## Consent

All participants provided written informed consent prior to enrollment in the randomised clinical trial.

## Conflicts of Interest

The authors declare no conflicts of interest.

## Data Availability

The data that support the findings of this study are available from the corresponding author upon reasonable request.
